# Editorial: Renal function and related biomarkers in cardiovascular risk assessment and prevention

**DOI:** 10.3389/fcvm.2022.1069629

**Published:** 2022-11-11

**Authors:** Gen-Min Lin, Dexter Canoy

**Affiliations:** ^1^Department of Medicine, Hualien Armed Forces General Hospital, Hualien, Taiwan; ^2^Department of Medicine, Tri-Service General Hospital, National Defense Medical Center, Taipei, Taiwan; ^3^Deep Medicine, Nuffield Department of Women's and Reproductive Health, University of Oxford, Oxford, United Kingdom

**Keywords:** biomarkers, cardiovascular risk, population study, prevention, renal function

Chronic kidney disease (CKD) is a leading risk factor in the development of cardiovascular disease (CVD) and is associated with higher risk of mortality (1). Therefore, the CVD risk reduction in those with CKD is of great importance. The identification of high-risk patients, prior to the occurrence of CVD, could permit the implementation and development of new preventative therapies. In patients with CKD, numerous biomarkers have been identified *via* research; however their applicability for routine clinical application remains to be fully evaluated. Moreover, there is a lack of reproducibility and standardization in research in the role of such measurements in cardiovascular risk. In addition, there is limited understanding of how such measurements may vary between populations. In the current Topic Research, we collected 6 high quality studies to highlight novel epidemiological research, in different population groups in order to examine the correlation between the CVD risk and renal biomarkers.

With regard to albuminuria and dipstick proteinuria, Liang et al. performed a meta-analysis for the prognosis in patients with heart failure, and they found a greater risk of all-cause mortality associated with dipstick proteinuria [HR: 1.54 (95% CI: 1.28, 1.84)], microalbuminuria (urine albumin-to-creatinine ratio (ACR) of 30 - 299 mg/g) [HR: 1.54 (95% CI: 1.23, 1.93)] and macroalbuminuria (urine ACR ≥ 300 mg/g) [HR: 1.76 (95% CI: 1.21, 2.56)]. In another paper, Park et al. performed an insurance data-registered longitudinal study in 124,691 Korean adults, and transient trace albuminuria was defined by a presence of trace (±) reaction on a dipstick test performed in the 2005/2006 or the 2007/2008 visit. The results were consistent to the findings of the meta-analysis performed by Liang et al. that those with transient trace albuminuria may have a greater risk of all-cause and CVD mortalities [HRs: 1.39 (95% CI: 1.01, 1.92) and 2.18 (95% CI: 1.08, 5.98), respectively]. According to these study findings, albuminuria of any degree can be considered as a useful biomarker and predictor of mortality.

With regard to serum uric acid (SUA) and CKD which were mutually affected (2), we collected two studies in this Research Topic to address the issue. Russo et al. studied the independent impact of SUA and CKD on mortality using the Uric Acid Right for Heart Health (URRAH) project (3), and revealed that the incidence rate for CVD mortality in 215,618 person-years of follow-up, stratified based on estimated glomerular filtration rate (eGFR >90, 60–90 and < 60 mL/min/1.73 m^2^) was higher in patients with hyperuricemia (SUA ≥5.9 mg/dL) and albuminuria (3.8, 22.1 and 19.1, respectively) as compared to those with only one risk factor or none (0.4, 2.8 and 3.1, respectively). In addition, for each SUA increase of 1 mg/dL, the risk for all-cause mortality increased by 10% even after adjustment for potential confounders including eGFR and the presence of albuminuria. In another study using the China Health and Nutrition Survey (CHNS) conducted by Li et al., hyperuricemia was defined as 7.0 mg/dL or greater for men and 6.0 mg/dL or greater for women. CVD risk was graded using the Framingham risk score (FRS) as low (FRS < 10%), medium (FRS 10–20%), or high (FRS >20%). The study uncovered a negative multiplicative interaction between hyperuricemia and CVD risk on CKD. The odds ratio (OR) between hyperuricemia and CKD in the low-CVD risk group was 5.51 (95% CI: 4.03, 7.52), followed by 3.64 (95% CI: 2.61, 5.09) in the medium-CVD risk group and 2.89 (95% CI: 2.12, 3.96) in the high-CVD risk group. Moreover, hyperuricemia and age had an additive effect on CKD, with a synergy index of 2.26 (95% CI: 1.45, 3.52). Based on these study findings, there were significant interplays between SUA, CKD and CVD risk, and the role of hyperuricemia as a crucial biomarker to predict CKD and CVD was emphasized.

For the matrix Gla protein (MGP) (4), a small vitamin K-dependent protein, which is a mineralization inhibitor in vascular calcification, Wei et al. firstly examined the association between urinary MGP and all-cause mortality in 776 randomly recruited Flemish subjects. For a doubling of urinary MGP, the risk of all-cause and CVD mortalities was found to increase [HRs: 1.31 (95% CI: 1.01, 1.69) and 2.05 (95% CI: 1.11, 3.79), respectively] with adjustment for covariates including eGFR and microalbuminuria. The study was consistent to the previous reports that both circulating and urinary MGP could be utilized as a useful biomarker to predict mortality (5).

Finally, Gao et al. investigated the association of eGFR with CVD and all-cause mortalities in 2,366 old-aged Chinese patients with acute myocardial infarction (AMI) who were rarely documented (1). The study revealed that compared with eGFR ≤ 63.02 mL/min/1.73 m^2^, patients with eGFR of 63.03–78.45, 78.46–91.50, >91.51 mL/min/1.73 m^2^ had lower risks of CVD mortality [HRs: 0.58 (95% CI: 0.38, 0.90), 0.61 (95% CI: 0.38, 0.99), and 0.48 (95% CI: 0.25, 0.90), respectively] and all-cause mortality [HRs: 0.64 (95% CI: 0.47, 0.88), 0.61 (95% CI; 0.42, 0.88), and 0.54 (95% CI: 0.35, 0.84), respectively]. The studies highlighted the role of eGFR on mortality even in the elderly population.

Taken together, the present Research Topic represents an important source of up-to-date information, covering many aspects of renal function and related biomarkers in assessment and prevention of CVD events. More comprehensive knowledge according to these discoveries may bring about new perspectives.

## Author contributions

Both authors listed have made a substantial, direct, and intellectual contribution to the work and approved it for publication.

## Conflict of interest

The authors declare that the research was conducted in the absence of any commercial or financial relationships that could be construed as a potential conflict of interest.

## Publisher's note

All claims expressed in this article are solely those of the authors and do not necessarily represent those of their affiliated organizations, or those of the publisher, the editors and the reviewers. Any product that may be evaluated in this article, or claim that may be made by its manufacturer, is not guaranteed or endorsed by the publisher.
